# Correlations Between Colloidal Stability and Peroxidase Activity of Prussian Blue Nanozymes in Salt Solutions

**DOI:** 10.1021/acs.jpcb.5c01256

**Published:** 2025-06-26

**Authors:** Tamás Péter, Dóra Takács, Dániel Viczián, Bojana Katana, Nizar B. Alsharif, István Szilagyi

**Affiliations:** † MTA-SZTE Lendület Biocolloids Research Group, Department of Physical Chemistry and Materials Science, Interdisciplinary Excellence Centre, 83415 University of Szeged, Szeged H-6720, Hungary; ‡ Institute of Condensed Matter and Nanosciences - Bio and Soft Matter, Université catholique de Louvain, Louvain-la-Neuve B-1348, Belgium; § Department of Chemistry, Lund University, Lund SE-22100, Sweden

## Abstract

Prussian blue (PB) nanozymes have emerged as durable enzyme-mimicking catalysts with broad applications across many fields. Practical uses often involve exposure to salinity that influences their colloidal and catalytic behaviors, yet the specific effects of ions on PB particles are underexplored. This study investigates how electrolyte type and concentration affect the colloidal stability and enzyme-like activity of PB nanozymes using monovalent (NaCl, KCl, CsCl) and multivalent ions (CaCl_2_, LaCl_3_). Electrophoresis and dynamic light scattering measurements revealed that both concentration and ion composition significantly affect stability with specific ion adsorption altering charge density and aggregation, consistent with the DLVO theory. Findings further indicate that higher ionic strengths compress the electric double layer, improving substrate accessibility and accelerating horseradish peroxidase (HRP)-like catalytic reactions. Remarkably, Cs^+^ ions substantially boost activity through their unique ability to disrupt water structure and integrate into PB’s lattice. These findings highlight the importance of considering ion specificity when designing PB-containing dispersions for optimal stability and catalytic performance.

## Graphical abstract

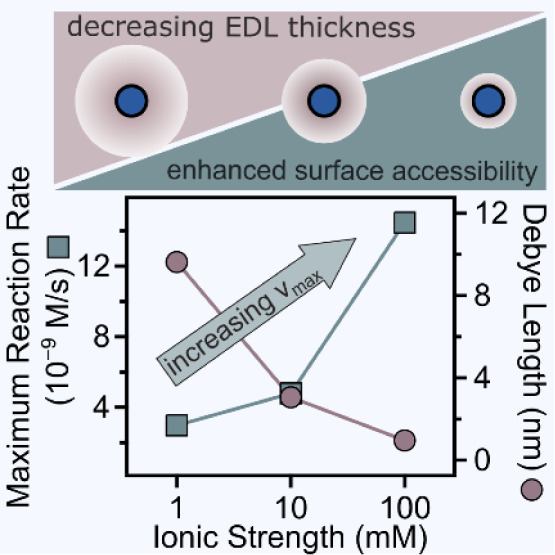

## 1 Introduction

Nanozymes, a class of enzyme-mimicking nanomaterials, have emerged as promising candidates in the rapidly growing field of biocatalysis.^1–4^ While the exact definition of nanozymes is still debated,^5^ their catalytic activity is strongly linked to several physical and chemical traits, including size, shape, composition, and surface characteristics.^6–8^ Unlike their biological enzyme counterparts, which are highly sensitive to the environment,^9^ nanozymes offer enhanced functional stability under a wider range of conditions. This resilience, combined with their cost-effective preparation and longer shelf life, makes nanozymes an attractive alternative to traditional enzymes in biomedical, environmental, and industrial applications.^10–12^ Recent advancements in the field have focused on single-atom nanozymes, which offer improved atomic utilization and tunable catalytic sites. Researchers are currently intensively investigating these systems and developing them for various biomedical applications, such as cancer therapy, antibacterial treatment, wound healing, and the management of neurological disorders.^13–15^

Nanozymes can function in complex biological fluids like blood or cellular cytoplasm, where maintaining colloidal stability is crucial, as it directly affects their catalytic efficiency, biodistribution, and biocompatibility.^16–19^ The colloidal and functional stability of particles in applications is contingent upon a number of parameters—i.e., surface charge and solvation effects^20–22^ —all of which are influenced by factors such as pH,^23^ temperature,^24^ and the surrounding components,^18,25–27^ similar to natural enzymes.^28,29^

Drawbacks of nanozymes include limited substrate specificity, lower catalytic efficiency compared to natural enzymes, potential toxicity, and challenges in practical applications.^11,30^ The latter limitation is related to nanozymes encountering diverse ions, which distinctly impact their colloidal and functional stability.^4^ Although research has systematically explored how different ions interact with the nanoparticles,^31–33^ there is still a lack of definitive quantitative data—such as aggregation constants, critical coagulation concentrations (CCC), or charge density information—specific to nanozymes. While numerous studies highlight the impact of the ionic environment on the activity of natural biocatalysts,^29,34–37^ similar analyses remain limited for nanozymes, despite the recognized importance of ion specificity. This gap in understanding how external factors, especially the presence of dissolved ions, influence nanozyme stability and activity underscores the need for further research in this area.

One compelling example of a nanozyme is Prussian blue (PB), a coordination polymer consisting of alternating ferric and ferrous ions coordinated by cyanide ligands.^16,38,39^ PB is a thermodynamically stable compound, and no structural degradation occurs even after long-term storage.^40^ PB particles not only mimic the functions of key antioxidant enzymes such as peroxidase, catalase, and superoxide dismutase but also offer chemical stability in biological environments.^16,41,42^ It was also demonstrated that not only surface iron atoms but also internal ones within PB participate in catalysis via electron transfer mechanisms.^43–45^ Particle size is critical in these systems as it directly influences catalytic efficiency and enables customization through synthesis conditions to optimize activity for specific applications,^46^ although aggregation processes can significantly affect the effective size. However, the mentioned research gap extends to PB particles, where in-depth studies of their colloidal behaviors in the presence of inorganic salts remain scarce. For example, a recent study demonstrated that NaCl-induced aggregation significantly affects the catalytic activity of PB nanoparticles, which highlights the importance of the correlation between colloidal stability and antioxidant activity.^47^ These findings suggest that it is warranted to investigate the effect of different ions as well in order to achieve a broader understanding of how electrolyte composition influences both colloidal stability and antioxidant activity, which is essential for developing robust and efficient PB-based nanozyme systems.

In the present study, therefore, the charging and aggregation properties of PB particles were studied in the presence of various electrolytes, and ion-specific effects on the interfacial behavior were explored, providing insight into how these factors affect colloidal stability and, consequently, the horseradish peroxidase (HRP)-like activity of the PB nanozymes.

## 2 Experimental Section

### 2.1 Materials

The chemicals used in the synthesis and the colloid analysis—acetone, K_3_[Fe­(CN)_6_], FeCl_2_·4H_2_O, NaCl, KCl, CsCl, CaCl_2_ and LaCl_3_—were purchased from VWR and used without further purification. Guaiacol used in the antioxidant tests was purchased from Acros Organics, and H_2_O_2_ (30%) was obtained from VWR. All solutions were prepared using ultrapure water (Adrona), and the pH was adjusted to 4 with HCl (VWR). The water as well as all the salt stock solutions were further filtered with 0.1 μm syringe filters (Millex) to avoid dust contamination.

### 2.2 Synthesis of PB Nanozyme

Prussian blue nanoparticles (PB) were prepared by coprecipitation. Initially, 100 mL of a 1 mM K_3_[Fe­(CN)_6_] solution was added dropwise to 100 mL of a 1 mM FeCl_2_ solution under vigorous stirring. Subsequently, 400 mL of acetone was added to the reaction mixture, resulting in dark blue precipitate formation. The resultant suspension was centrifuged at 2860 RCF for 30 min to separate the precipitate, which was washed three times with acetone and three times with distilled water to remove impurities. The purified PB was then dried overnight in a box furnace at 50 °C. From the resulting solids, a stock solution with a pH of 4 was prepared at a concentration of 1 g/L. The detailed structural characterization of the PB particles can be found elsewhere.^48^

### 2.3 Electrophoretic Light Scattering

Electrophoretic measurements were performed with a Litesizer 500 instrument (Anton Paar), equipped with a 658 nm wavelength laser source and a scattering angle of 175°. During sample preparation, appropriate volumes of salt solutions and water were mixed to achieve the desired ionic strength. PB particles were then added to the stock suspension, resulting in a particle concentration of 10 mg/L and a final volume of 2 mL. Samples were allowed to rest for 2 h at room temperature prior to each measurement, and the equilibration time in the instrument was 1 min. To characterize the surface charge of the particles, the electrophoretic mobilities were transformed into electrokinetic potentials ( ζ) utilizing the Smoluchowski equation.^49^ Following this, the surface charge density at the slip plane was calculated by fitting the potentials at various ionic strengths with the Debye–Hückel model, as outlined in the equation:^50^

1 $$\sigma =\varepsilon \varepsilon_{0}\kappa \zeta$$

where ε_0_ is the dielectric permittivity of the vacuum, ε is the dielectric constant of water, and *k* is the inverse Debye length, which involves the contribution of all ionic species in the electrical double layer. Accordingly, the concentration of the background electrolyte is included in the Debye length through the ionic strength­(*I*) as follows:

2 $$\kappa^{-1}=\sqrt{\frac{\varepsilon \varepsilon_{0}k_{B}T}{2N_{A}e^{2}I}}$$

where *K*_B_ is the Boltzmann constant, *T* is the absolute temperature,*N*_A_ is the Avogadro number, and *e* is the elementary charge. The thickness of the EDL can be estimated with the Debye length.

### 2.4 Dynamic Light Scattering

The hydrodynamic radius (*R*_h_) of the particles was determined by dynamic light scattering (DLS), utilizing an ALV-NIBS/HPPS particle sizer with a 633 nm laser source. The scattered light was collected at an angle of 173°, and a cumulant fit was used for data analysis.^51^ Correlation functions were collected for 20 s with 100 runs performed for each time-resolved experiment. For each measurement, 2 mL of dispersions were prepared in accordance with the methodology described above for the electrophoretic measurements, but the DLS experiments were initiated by the addition of the appropriate volume of the particle stock dispersions. The colloidal stability of the samples was quantified in terms of the stability ratio­(*W*) using the apparent aggregation rate coefficients (*k*_app_), which were obtained from time-dependent DLS experiments:

3 $$W=\frac{k_{app\left(fast\right)}}{k_{app}}=\frac{{\left(dR_{h}\left(t\right)/dt\right)}_{t\to 0}^{fast}}{{\left(dR_{h}\left(t\right)/dt\right)}_{t\to 0}}$$

where the subscript “fast” indicates diffusion-controlled aggregation of the particles. The value of *k*_app(fast)_ was determined separately in each system in the fast aggregation regime beyond the critical coagulation concentration (CCC) and was then used to calculate the stability ratios. The destabilization potential of a specific salt was determined using the CCC, at which the transition from rapid aggregation (*W* = 1) to a stable dispersion (*W* ≫ 1) occurs. This was calculated using the following equation:^52^

4 $$W=1+{\left(\frac{CCC}{c}\right)}^{\beta }$$

with *c* representing the molar concentration of the salt and the value of β was derived from the slope of the stability plots in the slow aggregation regime (i.e., before the CCC).

### 2.5 Horseradish Peroxidase Assay

The HRP assay is based on the oxidation of guaiacol substrate by H_2_O_2_ in the presence of horseradish peroxidase (HRP) or its mimic.^53^ The resulting tetraguaiacol, a brown compound with an absorption maximum of 470 nm, can be monitored by UV–vis spectrophotometry. The guaiacol concentration was varied between 1–12 mM, while the concentrations of H_2_O_2_ and the PB particles were kept at 2.6 mM and 10 mg/L, respectively. Since the aim was to investigate the correlation between colloid properties and enzymatic activity, the effects of different salts used during stability measurements were investigated at three different concentrations. First, at low salt concentrations, i.e., in the slow aggregation range (W > 100), at intermediate aggregation rates (W ∼ 10) and in the fast aggregation range (W ∼ 1). The pH of the stock solutions was adjusted to 4 by using HCl. The experiments were initiated following the addition of the particle stock, which was mixed with the sample for 10 s. The slopes of the absorbance–time graphs represent the corresponding reaction rates­(*v*) in 1/s, which were converted to mM/s units by applying the Beer–Lambert law. The optical light path is 1 cm, and the molar extinction coefficient of the tetraguaiacol is 26.6 mM^–1^ cm^–1^. The reaction rate was then plotted as a function of the guaiacol concentration­([*S*])­in the sample. The HRP-like activity was evaluated by fitting the plotted data with the Michaelis–Menten model of enzyme kinetics:^54^

5 $$v=\frac{v_{max}\left[S\right]}{K_{m}+\left[S\right]}$$

The maximum reaction rate (*v*_max_) represents the highest rate attainable under the given conditions. Further increases in substrate concentration do not result in additional increases in rate due to saturation of the catalytic sites of the enzyme or its mimics. The Michaelis–Menten constant (*K*_m_) refers to the substrate concentration (i.e., guaiacol) corresponding to half the *v*_max_. The error of this measurement protocol is within 5%.

## 3 Results and Discussion

### 3.1 Colloidal Stability Assessment

The salt composition was systematically varied in terms of the concentration, counterion type (NaCl, KCl, and CsCl), and ionic valence (NaCl, CaCl_2_, and LaCl_3_). Accordingly, the absolute value of the electrophoretic mobilities of PB decreased with the electrolyte concentration in each salt solution due to charge screening by the ions and remained close to zero at higher ionic strengths (Figure 1a,c). Although the PB particles were negatively charged throughout the concentration range studied, the exact mobility values—under a given experimental condition—differed significantly due to specific ion adsorption. This was further confirmed by the charge density values (Table 1), which were determined from the concentration-dependent mobility plots using eq 1, and followed the NaCl > KCl > CsCl order when investigating the monovalent ions, and the KCl > CaCl_2_ > LaCl_3_ order in the case of the ionic valence variation.

**Figure 1:**
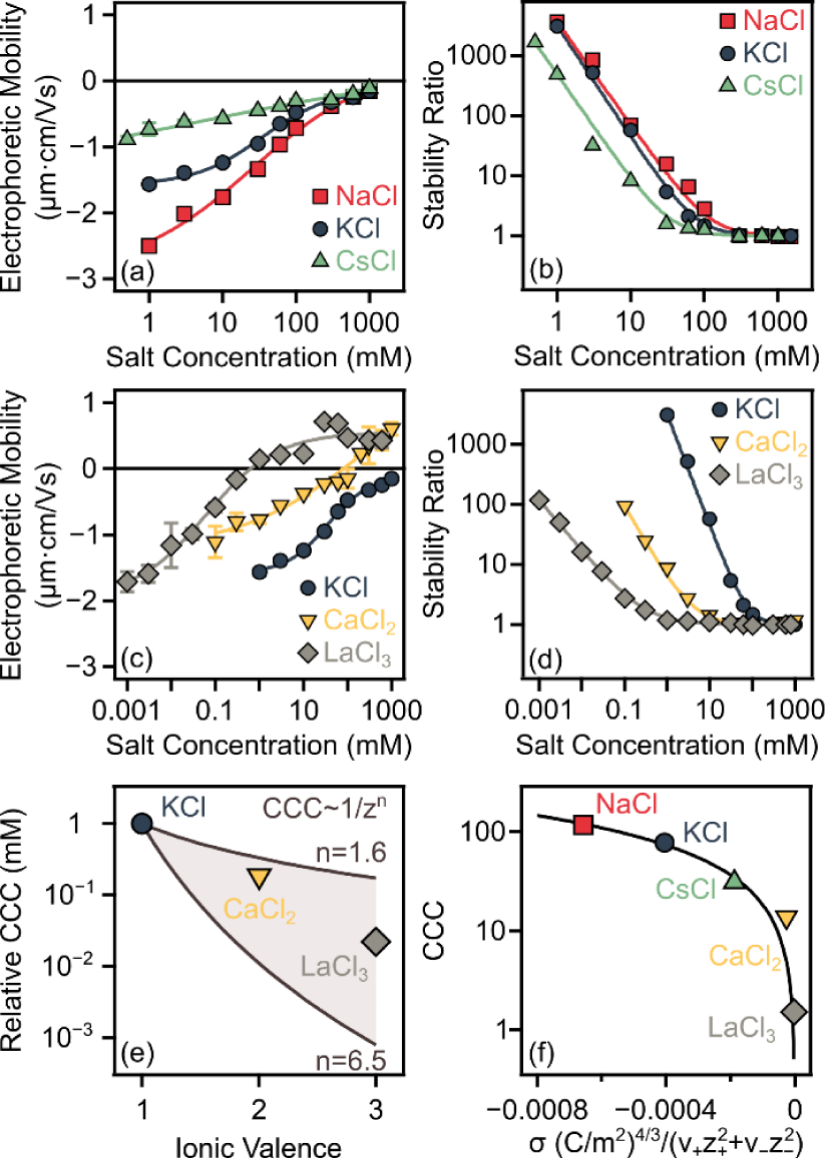
(a,c) Electrophoretic mobilities and (b,d) stability ratios of PB as a function of the salt concentration adjusted with different electrolytes. The solid lines for mobility data just serve to guide the eyes, while those for stability ratios were calculated with eq 4. (e) Relative CCC values (normalized to the CCC obtained in the presence of KCl) as a function of the ionic valence. The solid lines indicate the direct (for *n* = 1.6 and 6.5 in eq 6) Schulze–Hardy rules. (f) Dependence of the CCC on the charge density at the slip plane, which was normalized with the stoichiometric coefficient and the valence of the electrolytes. Data points refer to experimental CCC values, while the solid line was calculated by eq 7, showing a theoretical relation between surface charge density and CCC.

**Table 1:** Characteristic Charging and Aggregation Data of PB Particles with Different Salts^a^ ^b^

Salts	NaCl	KCl	CsCl	CaCl_2_	LaCl_3_
σ (mC/m^2^)^a^	–6.9	–4.8	–2.7	–1.4	–0.4
CCC (mM)^b^	118	77	34	13	2

a Surface charge density determined with eq 1.

b Critical coagulation concentration calculated by eq 4. The uncertainty of the CCC determination is about 10%.

The aggregation processes were followed under the same experimental conditions (e.g., particle concentration, pH, salt concentration range, and composition) as those used for electrophoresis, enabling direct comparison of the observed trends. The results in Figure 1b,d show that the samples were stable at low electrolyte concentrations, as indicated by the high stability ratio (eq 3) values, whereas at higher electrolyte concentrations, the dispersions became unstable, as stability ratios close to one were obtained. These two regimes are separated by the CCC, which is the parameter that can adequately describe the destabilization power of the given salts, and the obtained values followed the order NaCl > KCl > CsCl and KCl > CaCl_2_ > LaCl_3_ when examining the effect of ion specificity and ionic valence, respectively. These tendencies in the charging and aggregation features are typical for systems, in which the main interparticle forces originate from DLVO (Derjaguin, Landau, Verwey, and Overbeek)-type interactions such as van der Waals attraction and repulsion by the overlapping electrical double layers (EDL).^55,56^

The change in the CCC values was further explored through the Schulze–Hardy rule,^57^ which implies that the CCC dependence on the ionic valence (*z*) can be quantified as

6 $$CCC\propto 1/z^{n}$$

where the exponent *n* depends on the surface charge, the hydrophobicity of the particles, and the solvation level of the ions present in the solutions. For particles of low surface charge, the exponent is 1.6, while for highly charged particles, it is 6.5 when considering the valence of the counterions.^58^ In Figure 1e, the relative CCCs—i.e., CCCs normalized to the CCC obtained in the presence of KCl electrolyte—are shown with the CCC values expected from the Schulze–Hardy rule indicating the aforementioned limits. The results obtained for the divalent (Ca^2+^) and trivalent (La^3+^) counterions are in good quantitative agreement with the prediction of the rule, and the fact that the results appear between the limits indicates that the PB particles are moderately charged.

Subsequently, the aggregation mechanism was further explored by plotting the experimental CCC values against surface charge density (σ) data and comparing them to CCC values calculated using the DLVO theory as^31^

7 $$CCC=\frac{0.94}{N_{A}L_{B}{\left(H\varepsilon \varepsilon_{0}\right)}^{2/3}\left(\nu_{+}z_{+}^{2}+\nu_{-}z_{-}^{2}\right)}\sigma^{4/3}$$

where *N*_A_ is Avogadro’s number, *H* is the Hamaker constant, *L*_B_ is the Bjerrum length (0.72 nm at room temperature in water), *v*_+_ and *v_–_* are the stoichiometric coefficients, while *z*_+_and *z_–_* represent the ionic valences for cations and anions, respectively. A Hamaker constant of 1.8 × 10^–21^ J provided the best fit between the calculated and measured data. The good agreement between the experimental (data points) and theoretical (CCC versus charge density fit) results (Figure 1f) indicates that the colloidal stability of PB dispersions can be indeed described considering DLVO-type forces, balancing attractive van der Waals and repulsive EDL interactions. However, ion-specific adsorption significantly affects the surface charge density values and alters the strength of the repulsive double layer forces, resulting in different CCCs.

### 3.2 HRP-like Activity of PB Nanozymes

PB particles can mimic the function of several naturally occurring enzymes including HRP,^16,30^ which catalyzes the oxidation of various organic and inorganic substrates using H_2_O_2_.^59^ To test its HRP-like activity, the oxidation of the guaiacol substrate by H_2_O_2_ is followed in the presence of PB, which acts as a catalyst.^48,53^ During the reaction, guaiacol is converted to its oxidized brown form, allowing the oxidation process to be monitored using a UV–vis spectrophotometer. To address the effect of colloidal stability on the antioxidant activity of PB particles, the HRP assay was performed in the presence of various electrolytes at three different levels (Figure 2), namely, at low salt concentrations in the slow aggregation range (*W* > 100), at intermediate aggregation rates (*W* ∼ 10) and in the fast aggregation range (*W* ∼ 1). In all cases, the reaction rates increased in parallel with the substrate concentration until reaching a saturation plateau after which further increases in substrate concentration had no effect, indicating that all catalytic sites on the PB particles were saturated. The obtained experimental data points were fitted using the Michaelis–Menten equation (eq 5) to determine the maximum reaction rate (*v*_max_). In general, the calculated data agreed well with the experimental data, confirming the enzyme-like behavior of the PB nanozymes. For NaCl (Figure 2a), KCl (Figure 2b), CaCl_2_ (Figure 2d), and LaCl_3_ (Figure 2e), only a slight increase was observed in the reaction rates with rising electrolyte concentration, while CsCl (Figure 2c) induced a much more pronounced effect, yielding significantly higher *v*_max_ values even at 1 mM ionic strength compared to other salts (Figure 2f). These results clearly indicate the HRP-mimicking ability of PB and along with its superoxide dismutase-like activity reported earlier,^48^ PB could be considered an efficient antioxidant nanozyme.

**Figure 2:**
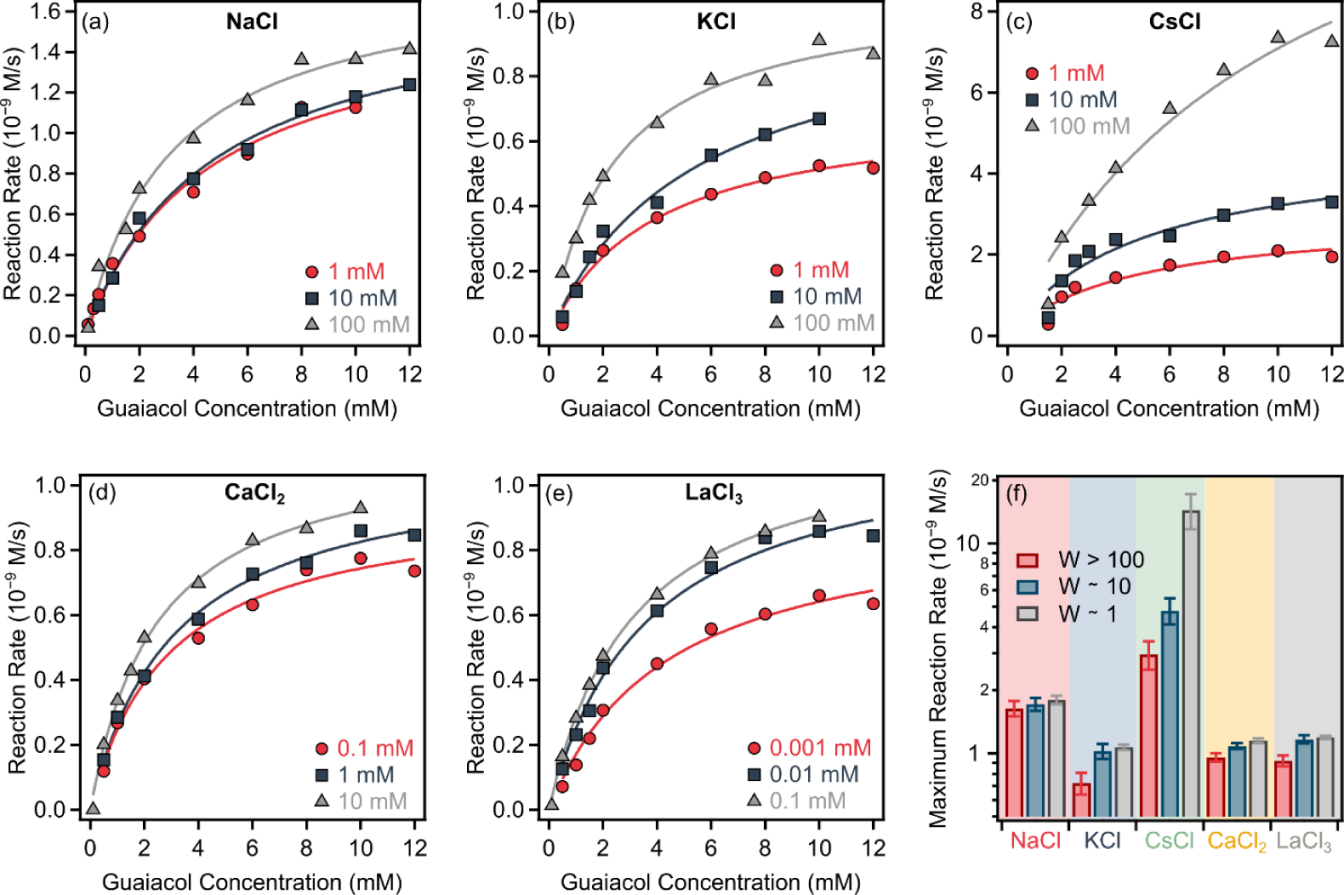
Change in reaction rate as a function of guaiacol concentration in the presence of different concentrations of NaCl (a), KCl (b), CsCl (c), CaCl_2_ (d), and LaCl_3_ (e). The lines correspond to fits based on the Michaelis–Menten model (eq 5). (f) shows the *v*_max_ values determined in systems with different stability ratios.

### 3.3 Correlation Between Interfacial Properties and Enzyme Mimicking Function

Overall, the data shown in Figure 2f indicate a clear correlation between the maximum reaction rate and the salt concentration, regardless of the specific salt composition. While particle aggregation is often seen as a hindrance to catalytic reactions due to its reduction of the availability of reactive centers, in this instance, the aggregation process was still in its early stages during the activity measurements. The HRP assay was conducted immediately after introducing PB particles into the system, meaning that aggregate formation did not significantly impact the observed trends and can be excluded from consideration in data interpretation.

The phenomenon can be attributed to the effect of ionic strength on the EDL in colloidal dispersions of charged particles. Accordingly, the increase in ionic strength decreases the thickness of the EDL—following the principles outlined in eq 2. Figure 3a,b shows that the maximum reaction rate increases as the EDL thins, corresponding to a shorter Debye length. At low ionic strength, the extended Debye length enhances electrostatic repulsion between similarly charged PB particles, promoting colloidal stability and reducing interactions with other polar entities such as the substrate (Figure 3c). In contrast, higher salt concentrations shorten the Debye length, allowing the substrate to approach the PB particle surface more closely, which may facilitate more favorable interactions with guaiacol and improve the catalytic process.

**Figure 3:**
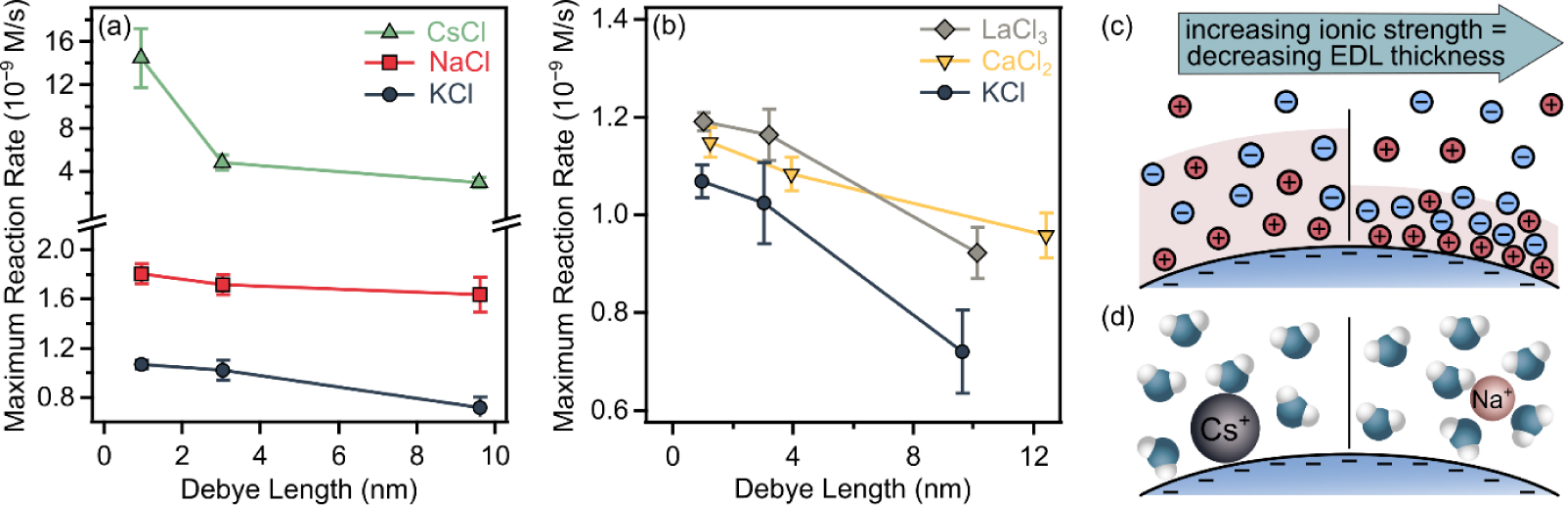
Dependence of the maximum reaction rate on the Debye length (calculated from eq 2) in the presence of counterions with the same (a) and different ionic valence (b). Schematic representation illustrating the effect of ionic strength (c) and ion specificity (d) on the structure of the EDL.

For monovalent cations, the order of maximum reaction rate at a given Debye length follows Cs^+^ > Na^+^ > K^+^, while for cations of different valences, it aligns as K^+^ < Ca^2+^ < La^3+^, consistent with the Schulze–Hardy rule. This order suggests that the ion specificity stems from how solvated cations influence water structure and hydrogen bonding, which are critical for proton–electron transfer reactions.^60,61^ Larger cations, such as Cs^+^, disrupt the water structure more than smaller ones, like Na^+^, as illustrated in Figure 3d. With larger cations, fewer water molecules remain at the surface due to partial desolvation, allowing closer interaction with the charged surface. This makes the hydration shell more permeable, facilitating the reactant interaction with the surface and potentially enhancing catalytic activity. In contrast, smaller cations retain more tightly bound hydration shells, leading to weaker surface interactions. This tendency is well described in studies examining the influence of monovalent cations on electrocatalytic reaction efficiency.^61,62^

The effect is particularly pronounced with Cs^+^ due to its strong affinity for PB particles, likely attributed to its ability not only to adsorb on the PB surface through electrostatic interactions but also to integrate into lattice defect sites by ion exchange.^63^ In addition, a recent study showed that Cs^+^-doped PB exhibited higher catalytic activity, as the presence of Cs^+^ promoted the generation of hydroxyl radicals (•OH).^64^ Notably, this enhanced •OH production was also observed when Cs^+^ ions were present in the medium, suggesting that, regardless of the exact mode of interaction, Cs^+^ ions facilitate radical formation. It is also worth noting that the reaction rate increases more significantly with higher ionic strength in the presence of Cs^+^ compared to other salts aligning with previous findings that higher Cs^+^ concentrations lead to enhanced adsorption on PB particle surfaces.^63,65^

## 4 Conclusions

In summary, our findings highlight how electrolyte concentration and ion specificity jointly influence the colloidal stability and HRP-like activity (i.e., catalytic oxidation of a substrate using H_2_O_2_) of PB particle dispersions. The DLVO theory and Schulze–Hardy rule successfully explain the trends in the charging and aggregation processes both qualitatively and quantitatively. The ion-dependent adsorption significantly affects the catalytic efficiency, as observed in the HRP-like activity in guaiacol oxidation across various electrolyte conditions. At higher salt concentrations, compression of the EDL enhances access of the substrate to the particle surface, increasing the reaction rate. Notably, Cs^+^ ions exhibit the most prominent effect, likely due to their ability to disrupt water structure and integrate into PB’s lattice, promoting higher catalytic activity. These findings demonstrate that electrolyte properties can substantially influence the colloidal and functional stability of PB particles by tuning noncovalent interactions and solvation environments.

## References

[1] Koley P., Jakku R., Hosseinnejad T., Periasamy S., Bhargava S. K. (2024). Immobilizing nanozymes on 3D-printed metal substrates
for enhanced peroxidase-like activity and trace-level glucose detection. Nanoscale.

[2] Zandieh M., Liu J. W. (2023). Nanozymes: Definition, activity, and mechanisms. Adv. Mater..

[3] Razlivina J., Dmitrenko A., Vinogradov V. (2024). AI-powered knowledge base enables
transparent prediction of nanozyme multiple catalytic activity. J. Phys. Chem. Lett..

[4] Halmagyi T. G., Noureen L., Szerlauth A., Szilagyi I. (2024). Engineering inorganic
nanozyme architectures for decomposition of reactive oxygen species. Dalton Trans..

[5] Wei H., Gao L. Z., Fan K. L., Liu J. W., He J. Y., Qu X. G., Dong S. J., Wang E. K., Yan X. Y. (2021). Nanozymes:
A clear definition with fuzzy edges. Nano Today.

[6] Zandieh M., Liu J. W. (2022). Surface science
of nanozymes and defining a nanozyme
unit. Langmuir.

[7] Huang Y. Y., Ren J. S., Qu X. G. (2019). Nanozymes:
Classification, catalytic
mechanisms, activity regulation, and applications. Chem. Rev..

[8] Bera D., Mukhopadhyay A., Nonappa N., Goswami N. (2023). *In
situ* depletion-guided engineering of nanoshell-like gold
nanocluster
assemblies with enhanced peroxidase-like nanozyme activity. J. Phys. Chem. Lett..

[9] Dinmukhamed T., Huang Z. Y., Liu Y. F., Lv X. Q., Li J. H., Du G. C., Liu L. (2021). Current advances in
design and engineering
strategies of industrial enzymes. Syst. Microbiol.
BioManuf..

[10] Wei H., Wang E. K. (2013). Nanomaterials
with enzyme-like characteristics (nanozymes):
Next-generation artificial enzymes. Chem. Soc.
Rev..

[11] Liang M. M., Yan X. Y. (2019). Nanozymes: From
new concepts, mechanisms, and standards
to applications. Acc. Chem. Res..

[12] Ma X. J., Lang J. Y., Chen P. Y., Tang W. J., Shindler S., Yang R. (2023). A cascade nanozyme
with antimicrobial effects against nontypeable
Haemophilus influenzae. Nanoscale.

[13] Zhang S., Zhang X.-D. (2024). Recent advances
in the bioactive structure and application
of single-atom nanozymes. Nano biomed. ENG.

[14] Zhang Y., Ya S., Huang J., Ju Y., Fang X., Ouyang X., Zeng Q., Zhou X., Yan X., Nie G. (2025). Spatial isolation of single copper­(I) sites
for cascade enzyme-like
catalysis and simultaneous ferroptosis/cuproptosis boosted immunotherapy. Exploration.

[15] Li Z., Fan X. W., Liu Y., Yue M. X., Wu T. T., Wang X., Jiang W., Fan K. L. (2025). Engineering mild-photothermal
responsive and NO donor Prussian Blue nanozymes using mild synthesis
for inflammation regulation and bacterial eradication in periodontal
disease. Adv. Mater..

[16] Estelrich J., Busquets M. A. (2021). Prussian Blue: A nanozyme with versatile catalytic
properties. Int. J. Mol. Sci..

[17] Ögüt E., Kip Ç., Gökçal B., Tuncel A. (2019). Aggregation-resistant
nanozyme containing accessible magnetite nanoparticles immobilized
in monodisperse-porous silica microspheres for colorimetric assay
of human genomic DNA. J. Colloid Interface Sci..

[18] Ju X. H., Fucíková A., Smíd B., Nováková J., Matolínová I., Matolín V., Janata M., Belinová T., Kalbácová M. H. (2020). Colloidal stability and catalytic
activity of cerium oxide nanoparticles in cell culture media. RSC Adv..

[19] Ghorbani M., Ercole F., Nazemi K., Warne N. M., Quinn J. F., Kempe K. (2024). A comparative study
on surface-engineered nanoceria using a catechol
copolymer design: colloidal stability vs. antioxidant activity. Nanoscale.

[20] Peula-Garcia J. M., Ortega-Vinuesa J. L., Bastos-Gonzalez D. (2010). Inversion of Hofmeister series by
changing the surface of colloidal particles from hydrophobic to hydrophilic. J. Phys. Chem. C.

[21] Li J., Lu N., Han S. P., Li X. M., Wang M. Q., Cai M. C., Tang Z. S., Zhang M. (2021). Construction of bio-nano interfaces
on nanozymes for bioanalysis. ACS Appl. Mater.
Interfaces.

[22] Nissler R., Dennebouy L., Gogos A., Gerken L. R. H., Dommke M., Zimmermann M., Pais M. A., Neuer A. L., Matter M. T., Kissling V. M. (2024). Protein aggregation on metal oxides governs
catalytic activity and cellular uptake. Small.

[23] Wang Q. Q., Chen J. X., Zhang H., Wu W. W., Zhang Z. Q., Dong S. J. (2018). Porous Co_3_O_4_ nanoplates with
pH-switchable peroxidase- and catalase-like activity. Nanoscale.

[24] Gao L. Z., Zhuang J., Nie L., Zhang J. B., Zhang Y., Gu N., Wang T. H., Feng J., Yang D. L., Perrett S. (2007). Intrinsic peroxidase-like activity of ferromagnetic nanoparticles. Nat. Nanotechnol..

[25] Rastogi L., Karunasagar D., Sashidhar R. B., Giri A. (2017). Peroxidase-like activity
of gum kondagogu reduced/stabilized palladium nanoparticles and its
analytical application for colorimetric detection of glucose in biological
samples. Sens. Actuators, B.

[26] Murath S., Alsharif N. B., Saringer S., Katana B., Somosi Z., Szilagyi I. (2020). Antioxidant materials
based on 2D nanostructures: A
review on recent progresses. Crystals.

[27] Ruiz-Cabello F. J. M., Oncsik T., Rodriguez-Valverde M.
A., Maroni P., Cabrerizo-Vilchez M. (2016). Specific ion effects and pH dependence on the interaction
forces between polystyrene particles. Langmuir.

[28] Ma X., Hortelao A. C., Patino T., Sanchez S. (2016). Enzyme catalysis to
power micro/nanomachines. ACS Nano.

[29] Salis A., Bilanicová D., Ninham B. W., Monduzzi M. (2007). Hofmeister effects
in enzymatic activity: Weak and strong electrolyte influences on the
activity of Candida rugosa lipase. J. Phys.
Chem. B.

[30] Ma X. Y., Zhang T. Y., Wang X. J., Zhang T. T., Zhang R. Y., Xu Z. H., Ma M. Z., Ma Y., Shi F. (2023). Nanoparticles
based on Prussian Blue for biosensor applications: A review. ACS Appl. Nano Mater.

[31] Yu W. Y., Du N., Gu Y. T., Yan J. G., Hou W. G. (2020). Specific ion effects
on the colloidal stability of layered double hydroxide single-layer
nanosheets. Langmuir.

[32] Rouster P., Pavlovic M., Szilagyi I. (2017). Destabilization
of titania nanosheet
suspensions by inorganic salts: Hofmeister series and Schulze-Hardy
rule. J. Phys. Chem. B.

[33] Oncsik T., Trefalt G., Borkovec M., Szilagyi I. (2015). Specific ion effects
on particle aggregation induced by monovalent salts within the Hofmeister
series. Langmuir.

[34] Lo
Nostro P., Ninham B. W. (2012). Hofmeister phenomena: An update on
ion specificity in biology. Chem. Rev..

[35] Park C., Raines R. T. (2001). Quantitative analysis
of the effect of salt concentration
on enzymatic catalysis. J. Am. Chem. Soc..

[36] Bauduin P., Renoncourt A., Touraud D., Kunz W., Ninham B. W. (2004). Hofmeister
effect on enzymatic catalysis and colloidal structures. Curr. Opin. Colloid Interface Sci..

[37] Yang Z., Liu X. J., Chen C., Halling P. J. (2010). Hofmeister effects
on activity and stability of alkaline phosphatase. BBA-Prot. Proteom..

[38] Zakaria M. B., Chikyow T. (2017). Recent advances in Prussian blue
and Prussian blue
analogues: Synthesis and thermal treatments. Coord. Chem. Rev..

[39] Chang S. Y., Hou W. T., Conde-Delmoral A., Ullah I., Gomez J. F. F., Morell G., Wu X. Y. (2025). A high-efficiency
and long-cycling
aqueous indium metal battery enabled by synergistic In^3+^/K^+^ interactions. Nanoscale.

[40] Mohammad A., Yang Y., Khan M. A., Faustino P. J. (2015). Long-term stability
study of Prussian blue-A quality assessment of water content and cyanide
release. Clin. Toxicol..

[41] Zhang W., Hu S. L., Yin J. J., He W. W., Lu W., Ma M., Gu N., Zhang Y. (2016). Prussian Blue nanoparticles as multienzyme
mimetics and reactive oxygen species scavengers. J. Am. Chem. Soc..

[42] Voros A., Halmagyi T. G., Saringer S., Hornok V., Szilagyi I. (2024). Papain functionalized
Prussian blue nanozyme colloids of triple enzymatic function. Chem. Commun..

[43] Komkova M. A., Ibragimova O. A., Karyakina E. E., Karyakin A. A. (2021). Catalytic pathway
of nanozyme “artificial peroxidase” with 100-Fold greater
bimolecular rate constants compared to those of the enzyme. J. Phys. Chem. Lett..

[44] Komkova M. A., Kostyukov A. A., Shneiderman A. A., Kuzmin V. A., Karyakin A. A. (2024). Fast reaction
of the Prussian Blue based nanozyme “artificial peroxidase”
with the substrates: Pre-steady-state kinetic approach. J. Phys. Chem. Lett..

[45] Komkova M. A., Karyakina E. E., Karyakin A. A. (2018). Catalytically synthesized
Prussian
Blue nanoparticles defeating natural enzyme peroxidase. J. Am. Chem. Soc..

[46] Khramtsov P., Kropaneva M., Minin A., Bochkova M., Timganova V., Maximov A., Puzik A., Zamorina S., Rayev M. (2022). Prussian blue
nanozymes with enhanced catalytic activity: Size tuning and application
in ELISA-like immunoassay. Nanomaterials.

[47] Panferov V. G., Zhang W. J., D’Abruzzo N., Wang S. H., Liu J. W. (2024). Kinetic
profiling of oxidoreductase-mimicking nanozymes: Impact of multiple
activities, chemical transformations, and colloidal stability. ACS Nano.

[48] Alsharif N. B., Samu G. F., Sáringer S., Muráth S., Szilagyi I. (2020). A colloid approach to decorate latex particles with
Prussian blue nanozymes. J. Mol. Liq..

[49] Delgado A. V., Gonzalez-Caballero F., Hunter R. J., Koopal L. K., Lyklema J. (2007). Measurement
and interpretation of electrokinetic phenomena. J. Colloid Interface Sci..

[50] Russel, W. B. ; Saville, D. A. ; Schowalter, W. R. Colloidal dispersions; Cambridge University Press, 1989.

[51] Holthoff H., Egelhaaf S. U., Borkovec M., Schurtenberger P., Sticher H. (1996). Coagulation rate measurements of
colloidal particles
by simultaneous static and dynamic light scattering. Langmuir.

[52] Grolimund D., Elimelech M., Borkovec M. (2001). Aggregation and deposition kinetics
of mobile colloidal particles in natural porous media. Colloid Surf. A.

[53] Maehly A. C., Chance B. (1954). The assay of catalases and peroxidases. Methods Biochem. Anal..

[54] Johnson K. A., Goody R. S. (2011). The original Michaelis constant:
Translation of the
1913 Michaelis-Menten paper. Biochemistry.

[55] Behrens S. H., Borkovec M., Schurtenberger P. (1998). Aggregation
in charge-stabilized
colloidal suspensions revisited. Langmuir.

[56] Sinha P., Szilagyi I., Ruiz-Cabello F. J. M., Maroni P., Borkovec M. (2013). Attractive
forces between charged colloidal particles induced by multivalent
ions revealed by confronting aggregation and direct force measurements. J. Phys. Chem. Lett..

[57] Overbeek J. T. G. (1980). The
rule of Schulze and Hardy. Pure Appl. Chem..

[58] Trefalt G., Szilagyi I., Tellez G., Borkovec M. (2017). Colloidal stability
in asymmetric electrolytes: Modifications of the Schulze-Hardy rule. Langmuir.

[59] Veitch N. C. (2004). Horseradish
peroxidase: a modern view of a classic enzyme. Phytochemistry.

[60] Huang B. T., Rao R. R., You S. F., Myint K. H., Song Y. Z., Wang Y. M., Ding W. D., Giordano L., Zhang Y. R., Wang T. (2021). Cation-
and pH-dependent hydrogen evolution and oxidation
reaction kinetics. JACS Au.

[61] Sebastián-Pascual P., Shao-Horn Y., Escudero-Escribano M. (2022). Toward understanding the role of
the electric double layer structure and electrolyte effects on well-defined
interfaces for electrocatalysis. Curr. Opin.
Electrochem..

[62] Khani H., Santiago A. P. R., He T. W. (2023). An interfacial
view of cation effects
on electrocatalysis systems. Angew. Chem. Int.
Ed..

[63] Lee I., Kim S. H., Rethinasabapath M., Haldorai Y., Lee G. W., Choe S. R., Jang S. C., Kang S. M., Han Y. K., Roh C. (2018). Porous
3D Prussian blue/cellulose aerogel as a decorporation
agent for removal of ingested cesium from the gastrointestinal tract. Sci. Rep..

[64] Wang G. C., Yu K. H., Ma J. Y., Dong H. J., Du W., Lu M. Z., Wu Y. F., Feng K. Z., Ma M., Zhang Y. (2024). A trinity strategy
to boost the catalytic efficiency of Prussian
Blue nanozyme for paper-based glucose sensing devices. ACS Appl. Nano Mater..

[65] Nguyen M. N., Yaqub M., Kim S., Lee W. (2021). Optimization of cesium
adsorption by Prussian blue using experiments and gene expression
modeling. J. Water Process. Eng..

